# Peptidoglycan Switches Off the TLR2-Mediated Sperm Recognition and Triggers Sperm Localization in the Bovine Endometrium

**DOI:** 10.3389/fimmu.2020.619408

**Published:** 2021-02-11

**Authors:** Ibrahim Fouad Elesh, Mohamed Ali Marey, Mohammed Ali Zinnah, Ihshan Akthar, Tomoko Kawai, Fayrouz Naim, Wael Goda, Abdel Rahman A. Rawash, Motoki Sasaki, Masayuki Shimada, Akio Miyamoto

**Affiliations:** ^1^ Global Agromedicine Research Center (GAMRC), Obihiro University of Agriculture and Veterinary Medicine, Obihiro, Japan; ^2^ Department of Pathology, Faculty of Veterinary Medicine, Damanhour University, Damanhour, Egypt; ^3^ Department of Theriogenology, Faculty of Veterinary Medicine, Damanhour University, Damanhour, Egypt; ^4^ Department of Microbiology and Public Health, Faculty of Veterinary Medicine and Animal Science, Bangabandhu Sheikh Mujibur Rahman Agricultural University, Gazipur, Bangladesh; ^5^ Graduate School of Integrated Sciences for Life, Hiroshima University, Higashihiroshima, Japan; ^6^ Department of Microbiology, Faculty of Veterinary Medicine, Alexandria University, Alexandria, Egypt; ^7^ Department of Basic Veterinary Science, Obihiro University of Agriculture and Veterinary Medicine, Obihiro, Japan

**Keywords:** peptidoglycan, sperm, endometrium, toll-like receptor, cluster of differentiation 44

## Abstract

In mammals, the uterine mucosal immune system simultaneously recognizes and reacts to most bacteria as well as allogenic sperm mainly through the Toll-like receptors (TLR)2/4 signaling pathway. Here, we characterized the impact of pathogen-derived TLR2/4 ligands (peptidoglycan (PGN)/lipopolysaccharide (LPS)) on the *immune crosstalk* of sperm with the bovine endometrial epithelium. The real-time PCR analysis showed that the presence of low levels of PGN, but not LPS, blocked the sperm-induced inflammatory responses in bovine endometrial epithelial cells (BEECs) *in vitro*. Immunoblotting analysis revealed that PGN prevented the sperm-induced phosphorylation of JNK in BEECs. Activation or blockade of the TLR2 system in the endometrial epithelium verified that TLR2 signaling acts as a commonly-shared pathway for PGN and sperm recognition. The impairment of endometrial sperm recognition, induced by PGN, subsequently inhibited sperm phagocytosis by polymorphonuclear neutrophils (PMNs). Moreover, using an *ex vivo* endometrial explant that more closely resembles those *in vivo* conditions, showed that sperm provoked a mild and reversible endometrial tissue injury and triggered PMN recruitment into uterine glands, while PGN inhibited these events. Of note, PGN markedly increased the sperm attachment to uterine glands, and relatively so in the surface epithelium. However, addition of the anti-CD44 antibody into a PGN-sperm-explant co-culture completely blocked sperm attachment into glands and surface epithelia, indicating that the CD44 adhesion molecule is involved in the PGN-triggered sperm attachment to the endometrial epithelium. Together, these findings demonstrate that, the presence of PGN residues disrupts sperm immune recognition and prevents the physiological inflammation induced by sperm in the endometrial epithelium *via* the MyD88-dependent pathway of TLR2 signaling, possibly leading to impairment of uterine clearance and subsequent embryo receptivity.

## Introduction

In all mammals, the endometrial mucosa has a unique ability to deal with pathogens, allogeneic spermatozoa, and semi-allogeneic embryos (1–3). To achieve this, it seems likely that the uterus is equipped with an efficient and strictly controlled mucosal innate immune system that can differentially respond to these various antigens (4). Endometrial epithelia express highly conserved specific pattern recognition receptors (PRRs) that can recognize pathogens through pathogen-associated molecular patterns (PAMPs), and tissue injuries through damage-associated molecular patterns (DAMPs) (5). Among PRRs, Toll-like receptors (TLRs) efficiently recognize virtually all bacteria or their PAMPs, and mount an early immune response, resulting in the expression of inflammatory mediators (6). Consequently, immune cells (mainly polymorphonuclear cells, PMNs) are attracted to the site of infection, ensuring phagocytosis of invading microorganisms or cell fragments (7, 8). Identifying these early immunological mechanisms in the endometrium during pathophysiological conditions will be paramount for optimal fertility.

Specifically, bacterial peptidoglycan (PGN) is a lipopeptide-polymer forming most of the dry weight of G +ve (90%) and G -ve (20%) bacteria, while lipopolysaccharide (LPS) composes the remaining portion of the G -ve bacterial cell wall and is a more potent endotoxin (9). LPS was detected in the blood and uterine fluid of cows with mild and severe endometritis (10), while no information is available about PGN levels in uterine lumen during clinical and subclinical endometritis in cows. Upon recognition, high levels of PGN/LPS activate the TLR2/4 pathway and trigger intracellular signal transduction cascades that stimulate deleterious effects on the host immune responses through the release of a potentially lethal array of inflammatory mediators (11). However, sustained low levels of PGN (LPS) could maintain chronic regulatory effects on the host immune balance, likely due to the secretion of anti-inflammatory cytokines (12, 13).

Mating or artificial insemination (AI) induces transient inflammatory reactions in the endometrium of several species and is characterized by the physiological influx of PMNs into the uterine lumen for clearance of bacteria, dead sperm and tissue debris (14). Like bacteria, sperm are phagocytized by PMNs either directly through cell-cell attachment or through neutrophil extracellular traps, which ensnare sperm and hinder their motility (15). Rapid removal of sperm is thought to prevent acquired immune responses against sperm in dams since it is critical for endometrial receptivity and pre-implantation embryo development (14). Our recent studies showed that active, but not heat-inactivated, sperm stimulated the transient mRNA expression of inflammatory genes in bovine endometrial epithelial cells (BEECs) (16) through triggering TLR2/4 signaling pathways (17) that increased PMN phagocytosis toward sperm *in vitro* (16). Moreover, in an *ex vivo* model, bovine sperm preferentially migrated to endometrial glands “*sperm sensors*” and initiated an inflammatory response associated with the presence of PMNs along with sperm (18).

Since the bovine endometrium recognizes PGN/LPS and sperm *via* the TLR2/4 pathways (5, 17), this study aimed to investigate competitive and pathophysiological interactions during co-exposure of the endometrial mucosa to sperm in the presence or absence of PGN/LPS. Our initial observations showed that only low levels of PGN, but not LPS, blocked sperm-induced inflammatory responses in BEECs *in vitro.* Moreover, recently it was reported that sperm interactions with the immunological defenses of the uterus were mediated mainly by the glandular epithelium (18). Accordingly, we used an *ex vivo* model of intact bovine endometrium explants to investigate the impact of very small quantities of PGN on the site and dynamics of sperm interactions with the uterine mucosa under conditions that more closely resemble those *in vivo*. To the best of our knowledge, this type of competitive *crosstalk* has not been described, and thus understanding its underlying mechanisms could have important translational implications in the context of reproductive mucosal immunology.

## Materials and Methods

### Ethical Approval

The Committee of Animal Experiments at Obihiro University of Agriculture and Veterinary Medicine approved all experimental protocols and methods (permit no. 27-74).

### Experimental Design

To describe the impact of pathogen-derived TLR2/4 ligands (PGN/LPS) on sperm-uterine *immune crosstalk*, we used both *in vitro* and *ex vivo* studies depicting possible various modes of exposure.

#### *In Vitro* Study

**Figure 1:**
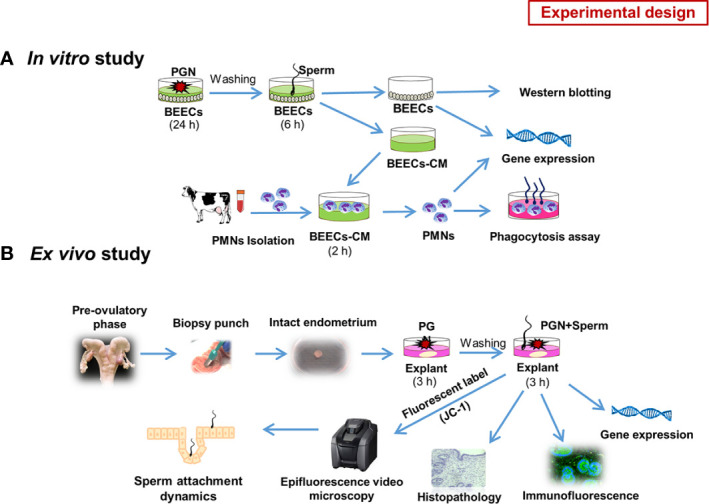
Schematic representation of the study experimental design: **(A)** *in vitro* model. Initially we investigated the competitive interaction of LPS/PGN and sperm with endometrial epithelium using RT-PCR. Based on the results, we gave more focus on the impact of low concentrations of PGN on sperm-induced inflammation in endometrial epithelium. As a main experiment, Subconfluent BEEC monolayers (90%) were stimulated with PGN (10 pg ml^−1^) for 24 h, followed by co-culture with sperm (5 × 10^6^ ml^−1^) for 6 h. The impact of PGN on sperm-induced immune responses in BEECs was assessed using RT-PCR. Later on, sperm-triggered MAPK cascade components in BEECs were assessed using western blotting. Conditioned medium (CM) from a sperm-BEEC co-culture after pre-exposure to PGN was harvested and exploited to challenge peripheral PMNs, isolated from mature healthy cows, for 2 h. Subsequently, PMN immune responses and phagocytic activity toward sperm were measured **(A)**. To assess direct BEEC response to PGN alone, BEECs were exposed to different concentrations of PGN at different time points. For activation or blockage of the TLR2 pathway, a TLR2 agonist (pam3Cys) or antagonist (CU-CPT22) was used, respectively. **(B)** *ex vivo* model. An explant model of intact endometrium was used to explore the sites of interactions and sperm dynamics with endometrial epithelium. We investigated immune responses and histomorphology induced by sperm in endometrial tissue in the presence of PGN. Additionally, we used high-resolution 3D imaging microscopy to trace sperm dynamics and attachment after JC-1 mitochondrial illumination of their mid-pieces **(B)**.

Initially, to investigate the competitive interaction of LPS/PGN and sperm with endometrial epithelium, subconfluent BEECs monolayers (90%) were stimulated with PGN (LPS) at high (100 and 1000 ng ml^−1^) or low (1, 10, and 100 pg ml^−1^) concentrations for 24 h, followed by co-culture with sperm (5 × 10^6^ ml^−1^) for 6 h. Based on the results, more focus was given to the impact of low concentrations of PGN on sperm-induced inflammation in BEECs. To assess the direct BEECs response to PGN alone, BEECs were exposed to different concentrations of PGN in a time dependent manner. Moreover, sperm-triggered MAPKs cascade components were assessed following pre-exposure to PGN. For activation or blockage of the TLR2 pathway, a TLR2 agonist (pam3Cys) or antagonist (CU-CPT22) was used, respectively. Conditioned media (CM) from a sperm-BEECs co-culture, after pre-exposure to PGN, was harvested and exploited to challenge peripheral PMNs isolated from mature healthy cows. Subsequently, PMNs immune responses and phagocytic activity toward sperm were measured. Guided by our previous investigations (16–18), we evaluated the mRNA transcripts of *TNFA, IL1B, IL8, PGES1, TLR2*, and *TLR4* as candidate inflammatory biomarkers throughout the entire study (**Figure 1A**).

#### *Ex Vivo* Study

The use of isolated BEECs monolayers may be compromised by the disruption of the endometrial architecture and by the release of DAMPs during tissue processing which may interfere with studying mechanisms of innate immunity and inflammation in the bovine endometrium (19). Therefore, an *ex vivo* model of intact bovine endometrium explants was also used to investigate the potential of PGN to affect the sites and dynamics of sperm interactions with the endometrial epithelium. Along with analyzing sperm-induced immune responses and changes in the architecture of endometrial tissues, a high-resolution 3D imaging microscopy was used to trace sperm dynamics and attachment after JC-1 mitochondrial illumination of their mid-pieces (**Figure 1B**).

### Uterine Sampling from Animals

Healthy pre-ovulatory bovine uteri were grossly confirmed free of any pathological lesions during routine slaughterhouse work (16). Fresh samples from animals were immediately transferred to the laboratory and treated under strict aseptic conditions for cell and organ cultures.

### Sperm Processing for Co-Culture

Semen straws were obtained from three highly fertile Holstein bulls belonging to Genetics Hokkaido Association, Hokkaido, Japan, where semen collection and processing were conducted under strictly controlled hygienic measures and the semen was routinely proven free from any bacterial contaminants. Semen straws were processed following a previously reported method (16). Briefly, nine straws (three straws from each bull) were thawed in a water bath at 38.5°C for 30 s, pooled, and washed three times using a Tyrode’s albumin, lactate, and pyruvate medium (Sp-TALP). A high-resolution 3D motion analysis of computer aided sperm analysis-sperm motility analysis system (*CASA-SMAS)* (DITECT, FHK, Tokyo, Japan) confirmed sperm motility >70%. In addition, the direct effect of natural and synthetic TLR2 agonists/antagonists on sperm motility was evaluated at different time points (0, 30, 60, and 120 min). Washed sperm were used for co-culturing with BEECs or intact endometrial explants.

### BEECs Culture

BEEC isolation and culture were performed according to a previously described protocol (16) with minor modifications. Cells were primed with pre-ovulatory levels of estradiol (E2) (1 ng ml^−1^) and progesterone (P4) (50 pg ml^−1^) until reaching confluence. Confluence and intact integrity were determined using area fraction (AF) output based on cellular translucence and boundaries (*ImageJ* software Version1.51j8). Epithelial cell purity >98% was evaluated using a monoclonal antibody against cytokeratin (anti-cytokeratin 8 + 18; ab53280, Abcam, Tokyo, Japan). Optimal viability of Annexin V-labeled cells was confirmed by FACS (Cell Sorter SH800S, Sony Biotechnology Inc., Bothell, WA, USA). Vimentin-negative staining indicated the absence of stromal cell contamination. Moreover, FACS analysis confirmed the absence of immune cell contamination using R-Phycoerythrin-conjugated mouse anti-CD45 hematopoietic cell marker (MA1–81458; Thermo Fisher Scientific, Waltham, MA).

### Pathogen-derived TLR2/4 Ligands

Ultrapure polymeric PGN from G +ve Staphylococcus aureus (InvivoGen, San Diego, CA, USA) and LPS from G -ve *Escherichia coli* O55: B5 (Sigma-Aldrich) were used as natural TLR2 and TLR4 ligands5 respectively, for treatments and co-culture.

### Activation and Blockage of TLR2 Pathway

For activation by the TLR2 agonist (17), subconfluent BEEC monolayers were pre-exposed to pam3Cys-Ser-(Lys)4, a synthetic analog of the triacylated N-terminal part of bacterial PGN (ab142085, Abcam) (10 pg ml^−1^), for 24 h followed by co-culture with sperm for 6 h. For blocking by the TLR2 antagonist (20), BEECs were pre-incubated with CU-CPT22, a synthetic TLR2 blocker (Merck, Darmstadt, Germany) (0.1 μM = 36.24 ng ml^−1^), for 3 h before being exposed to PGN (10 pg ml^−1^) for 24 h followed by co-culture with sperm for 6 h.

### Real-Time PCR

RNA was extracted from the cells using Trizol reagent (Invitrogen, Carlsbad, CA, USA) as previously reported (17). Extracted RNA was quantified using a NanoDrop Spectrophotometer 2000c (Thermo Scientific, Waltham, MA, USA), and the purity of each sample was assessed by the ratio A260/A280. The cDNA synthesis was done following the previously described protocol (21) with minor modifications. First, a DNase treatment step was performed using RQ1 RNase-Free DNase kit (Promega, Madison, WI, USA) to remove residual genomic DNA and other contaminants. The extracted RNA (1 μg) was incubated for 30 min at 37°C in a thermal cycler (Eppendorf, Hamburg, Germany) with a first mixture consisting of 1 μl of RQ1 RNase-free DNase 10× Reaction Buffer, 2 μl of RQ1 RNase-free DNase (1 unit/μl), and Nuclease-free water (Invitrogen, Carlsbad, CA, USA) to a final volume of 10 μl followed by addition of 1 μl of the RQ1 DNase Stop solution for 10 min at 65°C to terminate the reaction. The first-strand cDNA was synthetized according to the commercial protocol described in the SuperScript^®^ II Reverse Transcriptase kit (Invitrogen). Briefly, the DNase-treated RNA was incubated at 65°C for 5 min with a second mixture consisting of 1.5 μl of 3 μg/μl random primer, 1.5 μl of 10 mM PCR Nucleotide Mix (dNTP) (Roche Diagnostics, Indianapolis, IN, USA) and Nuclease-free water to a final volume of 18 μl. Next, a third mixture consisting of 6 μl of 5× First-Strand Buffer, 3 μl of 0.1M dithiothreitol, and 1.5 μl of 40 units/μl Ribonuclease Inhibitor Recombinant (Toyobo, Osaka, Japan), was added per each tube then, incubated at 42°C for 2 min followed by the addition of 0.2 μl of 200 units/μl SuperScript™ II Reverse Transcriptase and the thermal cycler was programmed at 25°C for 10 min, 42°C for 50 min, and 70°C for 15 min. The synthesized cDNA was stored at −30°C. The mRNA expression levels of our selected genes were determined by a quantitative real-time polymerase chain reaction (PCR) using an iQ5 real-time PCR detection system (Bio-Rad Laboratories, Tokyo, Japan). Simply, a total 10 µl reaction mix consisting of 2 µl/sample synthesized cDNA, 5 µl of QuantiTect SYBR Green PCR Master Mix (QIAGEN, Hilden, Germany), 0.2 µl of the targeted primer pairs (**Supplementary Table 1**), and 2.8 µl nuclease-free water (Invitrogen) was prepared. Then, the amplification program was run with an initial denaturation step at 95°C for 15 min, followed by 40 cycles of denaturation at 95°C for 15 sec, annealing at 51°C to 55°C (according to each primer) for 30 sec, extension at 72°C for 20 sec. The primers pairs were designed by Primer Express^®^ Software v3.0.1 (Thermo Scientific). The calculated cycle thresholds (Ct) were exported to Microsoft^®^ Office Excel, normalized using ACTB (β-actin) as a housekeeping gene, and the delta-delta comparative threshold method was used to quantify the fold change between the samples (22). All reactions were triplicated. Each run included a negative control (NCT) reaction, a non-reverse transcribed RNA (NRT) control, and a minus reverse transcriptase (MRT) control.

### Immunoblotting

BEECs were pre-exposed to PGN (10 pg ml^−1^) for 24 h followed by co-culture with sperm for 1 h. Briefly, protein samples from lysed BEECs were extracted by SDS polyacrylamide gel (10%) electrophoresis and blot probing following the antibody manufacturer’s instructions. Non-specific sites were blocked, and membranes were incubated with primary antibodies against target TLR2 downstream proteins triggered by sperm (17) **(** **Supplementary Table 2**). The bands’ intensities were analyzed using a Gel-Pro Analyzer (Media Cybernetics, Rockville, MD, USA).

### PMNs Cultivation

Mature PMNs from healthy animals were isolated as previously described (23). Propidium iodide (PI)-stained PMN viability was 99%, and population purity was >98% as assessed by FACS analysis. PMNs (7.0×10^6^ cells ml^−1^) were incubated in the sperm-BEEC CM for 2 h for determining PMN gene expression of pro- and anti-inflammatory cytokines.

### PMN Phagocytosis Assay

*In vitro* phagocytosis of sperm by PMNs challenged in sperm-BEEC CM was measured as detailed previously (23), with minor modifications. The rate of PMNs with engulfed sperm was regarded as the phagocytic index (PA). A count of 1000 PMNs with phagocytosed sperm was carried out independently by two observers.

### *Ex Vivo* Organ Explant Co-Culture

*Ex vivo *organ explants of endometrium from pre-ovulatory uteri were cultured according to a previously detailed protocol (18, 19), with minor modifications. Fresh, intact endometrial explants were pre-exposed to PGN (10 pg ml^−1^) for 3 h followed by co-culture with sperm for 3 h. Microscopically, examined explants maintained typical histomorphology and adequate tissue viability as confirmed using hematoxylin and eosin (H&E) tissue sections (24) and the mRNA expression of apoptotic *caspase 3* (25).

### Histopathology

Fixed specimens were serially dehydrated with ethanol, cleared into xylene shifts, and paraffinized again at 65°C as part of the routine FFPE technique previously detailed (26). Sections (3 μm thickness) from *ex vivo *organ explants of endometrium were stained H&E. Semi-quantitative scoring of histomorphology alterations was conducted in a blinded way in 10 fields selected randomly from each slide for each animal as described previously (27).

### Immunostaining

Immunostaining was used for assessment and site-specific localization of target inflammatory (TNFA) and signaling (TLR2) biomarker protein expression, as previously described (17), with minor modifications. Briefly, sections (3 μm) were incubated with primary antibodies against each protein target (**Supplementary Table 3**). Negative (omitted primary antibody) and isotype-matched (isotype-IgG) tissue controls were compared to reveal non-specific binding and false-positive expression. A high-resolution 3D scanning system combined with an all-in-one fluorescence microscope (Keyence, BZ-X800, Osaka, Japan) was used to localize the distribution of TNFA and TLR2 proteins in the endometrial tissue. The BZ-X GFP (OP-87763), BZ-X RED (OP-87765), and BZ-X DAPI (OP-87762) filters were set for green, red, and blue wavelengths, respectively. The mean fluorescence intensity (MFI) and integrated density (IntDen) analyses of arbitrarily selected fields were corrected into total cell fluorescence (CTCF) values after subtraction of background fluorescence for semi-quantitative scoring (28).

### Imaging of JC-1–labeled Sperm

*Ex vivo* organ explants of the endometrium were pre-exposed to PGN/pam3Cys (10 pg ml^−1^) for 3 h. Sperm were directly pre-incubated with the JC-1 mitochondrial stain (AdipoGen, San Diego, CA, USA) (at 6.4 μM) for 15 min for sperm labeling and visualization in explants (18). JC-1–labeled sperm (5 × 10^6^) were co-cultured with PGN-exposed explants and sperm behavior and localization were traced within 5 to 30 min. A fully-focused 3D high-resolution imaging using an all-in-one epifluorescence microscope (Osaka, Japan) equipped with a temperature-controlled thermal plate (TPi-SQX, Tokai Hit, Japan) was used to view the sperm dynamics and localization in explants. During recording, the focus was adjusted in order to visualize all sperm attached to the glands and surface epithelium. Videos recorded at 30 min were assessed for numbers of sperm localized in uterine glands and in surface epithelia using *ImageJ* software (Version 1.51j8) (18).

### Measuring Cluster of Differentiation 44 (CD44) mRNA Expression and Fluorescence Intensity in Bovine Endometrium

The RT-PCR assay was exploited to test the possibility of endometrial cells expressing the cellular adhesion molecule (CD44) in BEECs and explants. An immunofluorescence assay using rabbit polyclonal anti-CD44 antibody (1:50, 15675-1-AP, Proteinintech, USA) (**Supplementary Table 3**) was used to confirm the accumulation of CD44, especially in endometrial glands.

### CD44 Blocking Assay

Endometrial explants were exposed to the anti-CD44 neutralizing antibody (1:500) (28) for 3 h, washed, exposed to PGN (10 pg ml^−1^) for 3 h and then co-cultured with JC-1–labeled sperm. Recorded videos within 30 min were analyzed for sperm numbers associated with glands and surface epithelium using *ImageJ* software. Similarly, BEECs were exposed to the anti-CD44 antibody for 3 h, washed, exposed to PGN (10 pg ml^−1^) for 24 h, and then co-cultured with sperm for 6 h followed by the RT-PCR assay for detection of tested genes.

### Statistics

We designated the animal as the statistical unit, and data from at least three to four replicated experiments were presented as mean ± SEM. GraphPad Prism 8.0 software (La Jolla, CA, USA) was used to analyze differences among groups by performing one-way analysis of variance (ANOVA) followed by Bonferroni’s post-comparison test (>two groups) or two sample *t-test* (two groups) with significant differences at (*P<0.05, **P<0.01, or ***P<0.001).

## Results

### Low Concentrations of PGN, But Not LPS, Suppressed Sperm-induced Inflammatory Responses in BEECs

**Figure 2:**
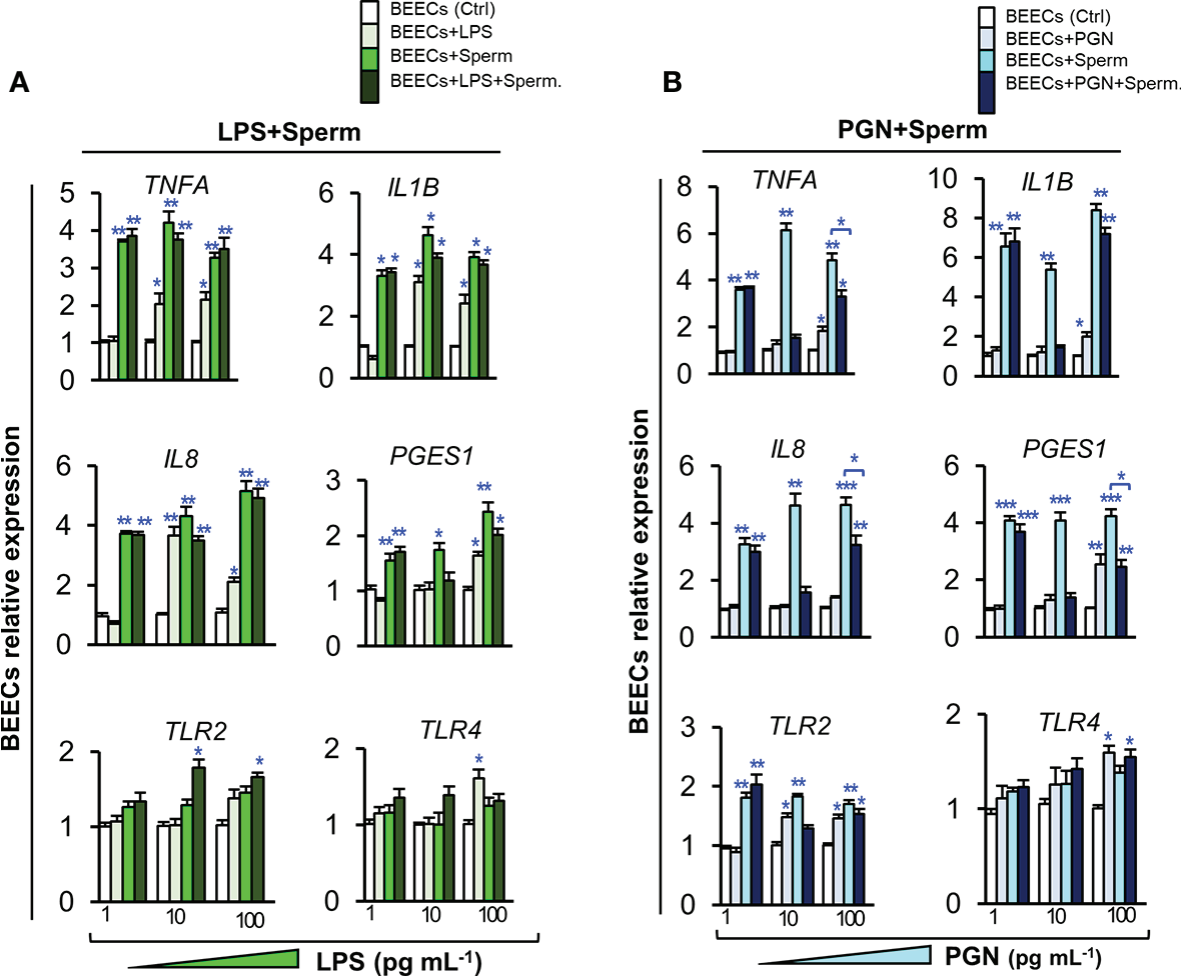
Low levels of PGN, but not LPS, suppressed the stimulatory effect of sperm on the transcription of major inflammatory cytokines, chemokine, *PGES1* and *TLR2* in BEECs. Subconfluent BEEC monolayers were exposed to **(A)** LPS (1, 10, and 100 pg ml^−1^), or **(B)** PGN (1, 10, and 100 pg ml^−1^) for 24 h followed by co-culturing with sperm (5 × 10^6^ cell ml^−1^) for 6 h. The mRNA transcription levels of major inflammatory genes (*TNFA, IL1B, IL8, PGES1, TLR2*, and *TLR4*) were quantified by real-time PCR assay. The animal was designated as the statistical unit, and the data was obtained from 3 replicated independent experiments, using BEECs from 3 different uteri, (3 wells/treatment/experiment) presented as mean ± SEM. Asterisks denote a significant variance (*P < 0.05 **P < 0.01, or ***P < 0.001) between the different treatment groups when compared to the control group at each PGN/LPS concentration. Asterisk denotes a significant variance (*P < 0.05) between the ‘Sperm’ and ‘PGN+Sperm’ groups.

To investigate the competitive interaction of PGN/LPS and sperm with endometrial epithelium, BEECs were exposed to PGN/LPS at high (100, and 1000 ng ml^−1^) or low concentrations (1, 10, and 100 pg ml^−1^) for 24 h followed by addition of sperm for further 6 h. The results showed that co-culturing of sperm with BEECs increased the mRNA expression of inflammatory cytokines (TNFA and IL1B), inflammatory chemokine (IL8), and PGES1 in BEECs. Moreover, high concentrations of LPS, either independently or combined with sperm, significantly increased transcription levels of tested genes in BEECs compared to sperm only (**Supplementary Figure 1A**). Additionally, the pre-exposure of BEECs to low levels of LPS induced inflammatory responses in BEECs, but do not interfere with the subsequent sperm-induced inflammation in BEECs (**Figure 2A**). High concentrations of PGN increased transcription levels of the tested genes in BEECs **(** **Supplementary Figure 1B** **)**. Nonetheless, low concentrations of PGN failed to trigger any significant inflammatory responses in BEECs (except for PGN 100 pg ml^−1^), they suppressed the sperm-induced abundance of expressions of inflammatory genes in BEECs, and this effect was maximum at PGN (10 pg ml^−1^) (**Figure 2B**).

### PGN-induced Immediate and Transient Inflammatory Responses in BEECs

**Figure 3:**
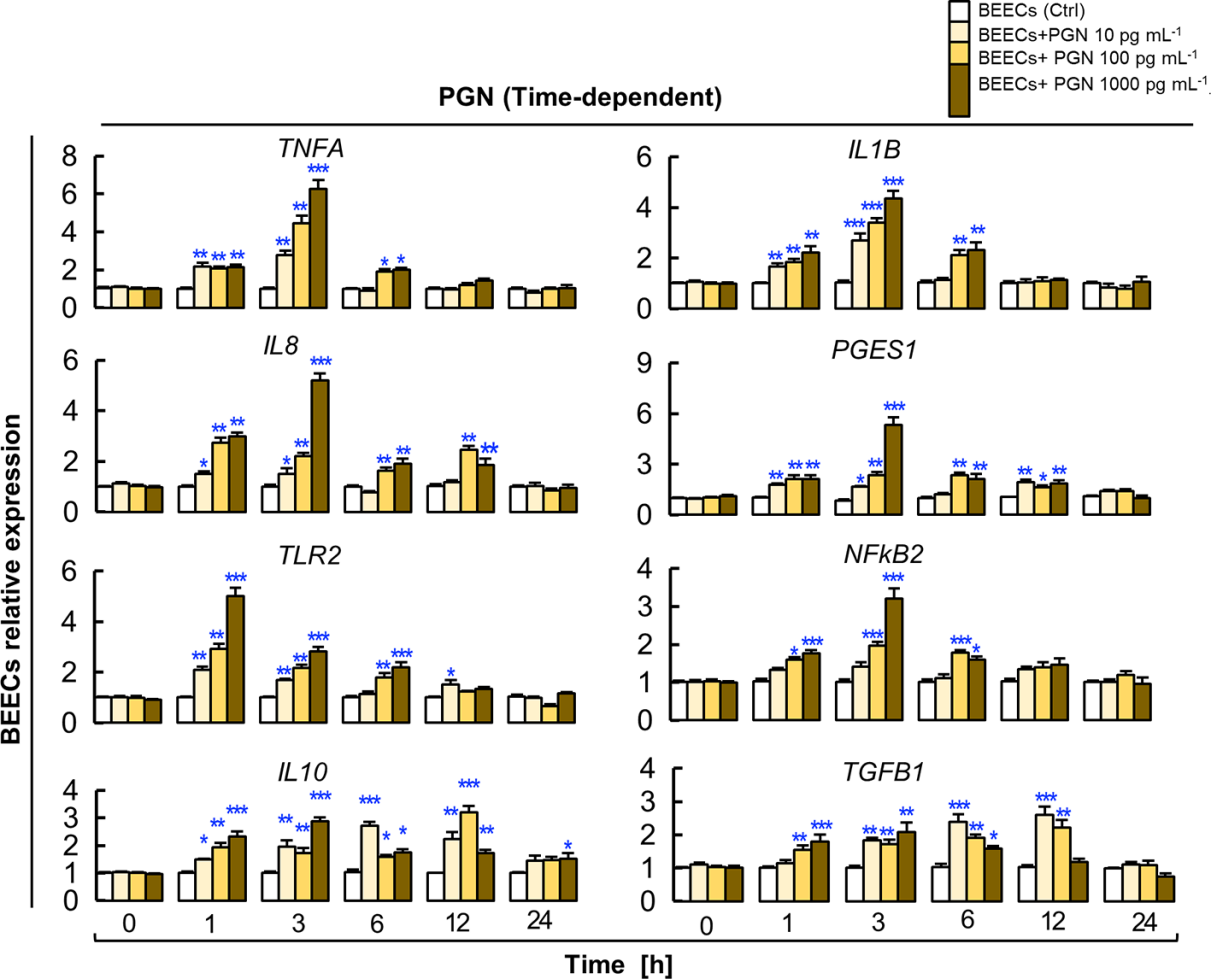
PGN induces transient upregulation of the transcription of major pro- and anti-inflammatory markers in BEECs. Subconfluent BEEC monolayers were exposed to different picogram concentrations (10, 100 and 1000 pg ml^−1^) of PGN at different time points (0, 1, 3, 6, 12, and 24 h). The mRNA expression of major pro- and anti-inflammatory markers was assessed using RT-PCR assay. Data are presented as mean ± SEM of 3 independent experiments using epithelial cells from 3 different uteri (3 wells/treatment/experiment). Asterisks denote a significant variance (*P < 0.05 **P < 0.01, or ***P < 0.001) between the different groups of PGN concentrations (pg/ml) when compared to the control group (0 pg/ml) at each time point.

Here, we investigated the impact of direct exposure to PGN (10, 100 and 1000 pg ml^−1^) on kinetics of inflammation in BEECs at different time points (0, 1, 3, 6, 12 and 24 h). The results showed that PGN, in a dose-dependent manner, started to upregulate levels of transcripts of the tested genes after 1 h, then the response reached its peak at 3 h, it started to subside after 6 h, and completely subsided after 12 to 24 h. Additionally, PGN consistently maintained high transcription levels of *IL10* and *TGFB1* genes at 1, 3, 6, 12 h and subsided at 24 h (**Figure 3**).

### PGN Blocked Sperm-triggered JNK Phosphorylation as a Downstream Target of the TLR2 Signal Transduction Cascade in BEECs

**Figure 4:**
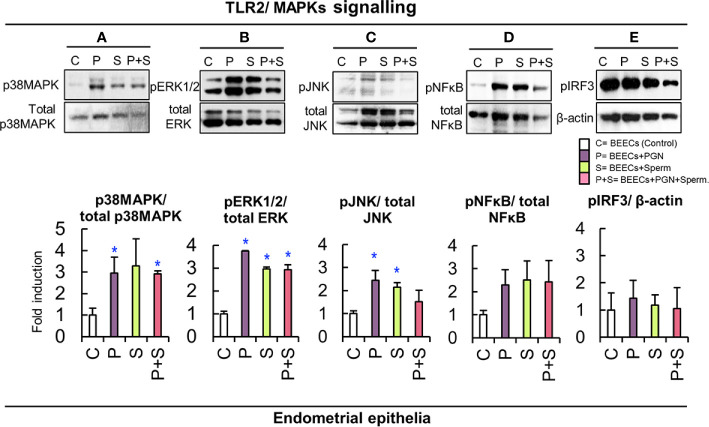
PGN combination perturbed the sperm-triggered JNK phosphorylation in endometrial epithelia. Subconfluent BEECs monolayers were exposed to PGN (10 pg ml^−1^) for 24 h followed by co-culturing with sperm for 1 h. Western blot analysis was carried out to estimate the phosphorylation levels of the mitogen-activated protein kinase (MAPK) cascade components in endometrial cells. Expression of specific antibodies against **(A)** P38 mitogen-activated protein kinase (p38MAPK), **(B)** phosphorylated extracellular signal-regulated kinase (pERK1/2), **(C)** c-Jun N-terminal kinase (JNK), **(D)** nuclear factor kappa-light-chain-enhancer of activated B cells (NF*κ*B), **(E)** phospho Interferon Regulatory Factor 3 (pIRF3) was assessed, and β actin was used as a loading control in the different groups. The value under each sample indicates the fold change of the protein level relative to that of the control. The bands’ intensity was analyzed using a Gel-Pro Analyzer (Media Cybernetics, Rockville, MD, USA). Data are presented as mean ± SEM of 3 independent experiments using epithelial cells from 3 different uteri (3 wells/treatment/experiment). Asterisks denote a significant variance (*P<0.05) between the different groups when compared to the control group.

Ligation of TLR4 receptors to their specific agonist stimulates two distinct pathways, MyD88-dependent pathway and TRIF-dependent pathway while, ligation of TLR2 stimulates MyD88-dependent pathway only (11). In our study, we focused on phosphorylation levels of p38MAPK, ERK1/2, and JNK as downstream targets of MyD88-dependent signaling pathways and IRF3 as downstream targets of TRIF-dependent signaling pathways. BEECs were pre-exposed to PGN (10 pg ml^−1^) for 24 h, washed, and followed by co-culturing with sperm for 1 h. Then, we analyzed phosphorylation levels of p38MAPK, ERK1/2, JNK, and IRF3 as downstream targets of TLR2/4 signaling pathways triggered by sperm in BEECs (17). Immunoblotting analysis revealed that either PGN or sperm stimulated the phosphorylation levels of p38MAPK, ERK1/2, and JNK in BEECs, while PGN combined with sperm prevented the sperm-induced phosphorylation of the JNK component in BEECs. Neither sperm nor PGN stimulated the phosphorylation of the IRF3 in BEECs (**Figure 4**).

### Activating TLR2 Pathway Blocked the Sperm-induced Inflammatory Response in BEECs

**Figure 5:**
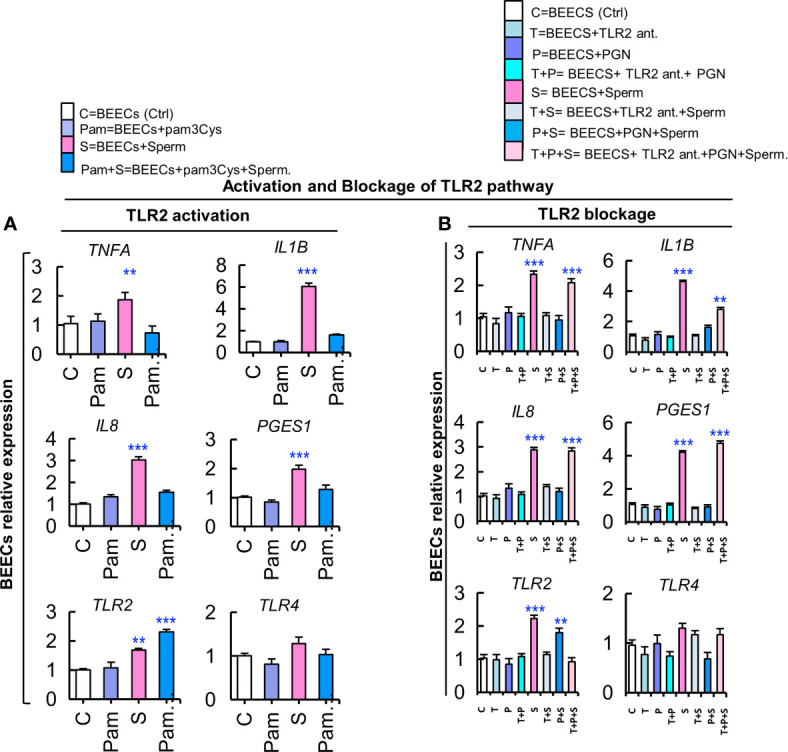
Activation of the TLR2 pathway suppresses the stimulatory effect of sperm on transcription of inflammatory cytokines, while a TLR2 blockade removed the PGN suppressive effect on sperm-induced inflammation in BEECs. **(A)** BEECs activation with pam3Cys (synthetic TLR2 agonist). Subconfluent BEEC monolayers were exposed to pam3Cys (10 pg ml^−1^) for 24 h followed by co-culturing with sperm for 6 h. **(B)** Blockage of TLR2 with synthetic TLR2 antagonist. BEECs were pre-exposed to CU-CPT22 (0.1 μM = 36.24 ng ml^−1^) for 3 h followed by exposure to PGN (10 pg ml^−1^) for 24 h before co-culturing with sperm for 6 h. The mRNA expression of major inflammatory genes was assessed using RT-PCR assay. Data are presented as mean ± SEM of 3 independent experiments using epithelial cells from 3 different uteri (3 wells/treatment/experiment). Asterisks denote a significant variance (*P<0.05 **P<0.01, or ***P<0.001) between the different groups when compared to the control group.

The above-mentioned results prompted us to propose that TLR2 signaling pathway is the common recognition system for PGN and sperm recognition in BEECs. To elucidate that, BEEC monolayers were exposed to synthetic TLR2 agonist (pam3Cys; 10 pg ml^−1^) for 24 h followed by co-culturing with sperm for 6 h. Pam3Cys fully blocked BEEC mRNA expressions of inflammatory mediators in response to sperm (**Figure 5A**) in a similar pattern to PGN, and this could further confirm that TLR2 is the central pathway for sperm recognition.

### Blocking the TLR2 Pathway Removed the PGN-Suppressive Effect on BEECs Inflammatory Responses to Sperm

Likewise, we used a synthetic TLR2 antagonist (CU-CPT22) to block the TLR2 pathway in BEECs. BEEC monolayers were exposed to CU-CPT22 for 3 h, washed, and exposed to PGN for 24 h before co-culturing with sperm for 6 h. Interestingly, the TLR2 blocker recovered the sperm-triggered transcription of tested genes that was suppressed by PGN (**Figure 5B**). These findings could imply that TLR2 signaling could be a commonly-shared pathway for PGN and sperm recognition in BEECs.

### PGN Suppressed the Stimulatory Impact of Sperm-BEECs Conditioned Medium on PMN Gene Expression and Phagocytic Behavior

**Figure 6:**
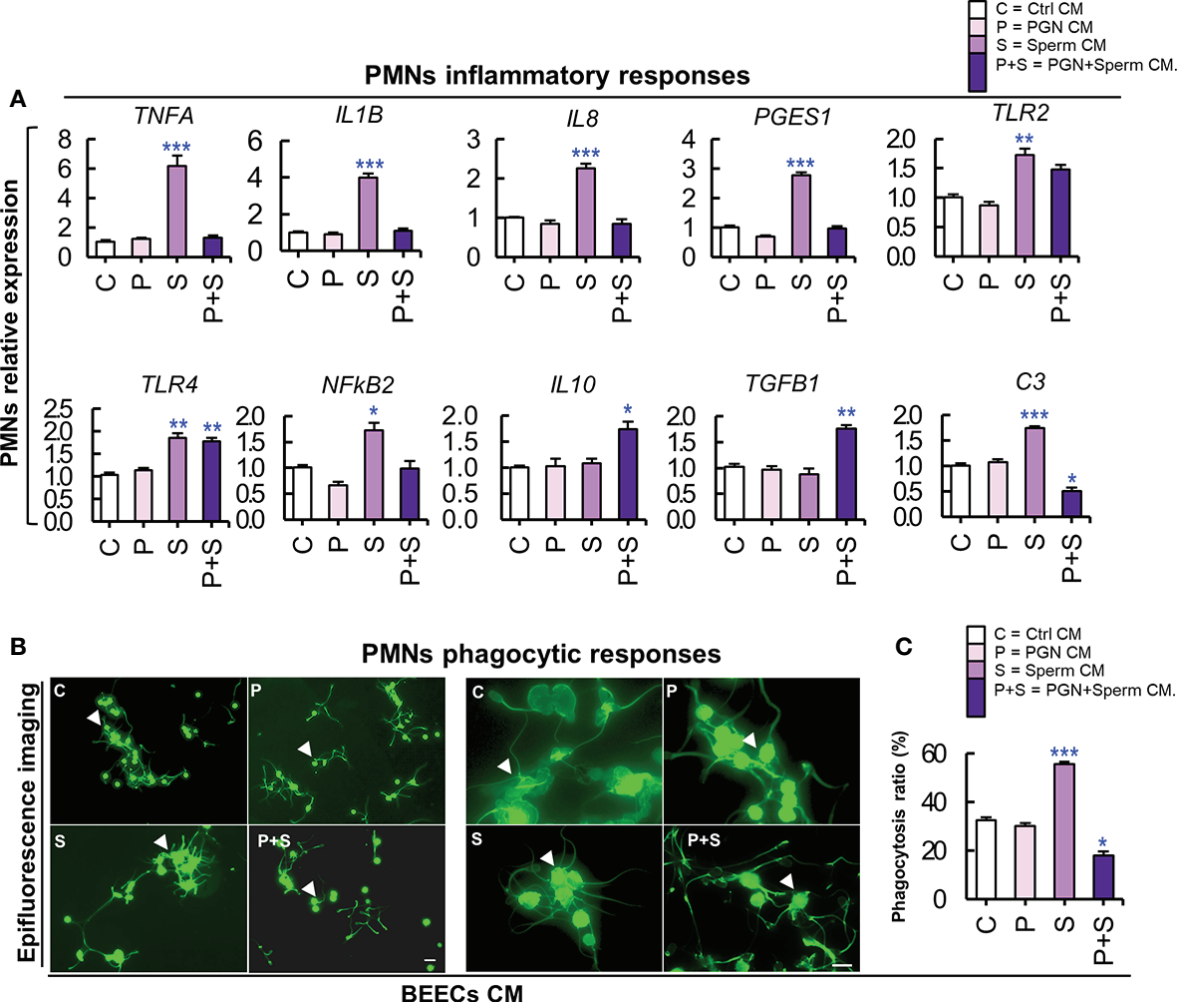
**(A)** PGN suppressed the stimulatory impact of sperm-BEECs conditioned media on the transcription of major pro-and anti-inflammatory genes of PMNs *in vitro*. Mature PMNs (7×10^6^ cells ml^−1^) isolated from circulating blood of healthy cows were challenged for 2 h in CM obtained from BEECs, exposed to PGN (10 pg ml^−1^) for 24 h, washed, and co-cultured with sperm for 6 h. The mRNA expression of different genes was quantified using RT-PCR assay. Data are presented as mean ± SEM of 3 independent experiments where asterisks denote a significant variance (*P<0.05 or **P<0.01) between the different groups when compared to the control group. **(B)** PGN downregulated the stimulatory impact of sperm-BEEC conditioned medium on phagocytic activity of PMNs. Similarly, mature PMNs (7×10^6^ cells ml^−1^) were challenged in this CM for 2 h followed by co-culture with sperm (7×10^6^ cells ml^−1^) for 1 h and phagocytosis was assessed. **(B)** Representative images of PMN phagocytosis for sperm. Magnification: 200× (left? panel) and 400× (right panel). (Control; C, PGN; P, sperm; S, and PGN + sperm; P+S). Arrow heads indicate PMNs phagocytosing sperm. Scale bar = 20 μm. **(C)** The phagocytosis ratio was independently assessed by two observers for 1000 cells using an all in one epifluorescence high-resolution imaging system (Keyence, BZ-X800, Osaka, Japan). Results are representative of 3 independent trials using 3 different healthy animals. Data are presented as mean ± SEM with asterisk denoting a significant variance (*P<0.05) between the different groups when compared to the control group. Asterisk denotes a significant variance (*P<0.05) between the ‘Sperm’ and ‘PGN+Sperm’ groups.

Our previous studies have documented that sperm-BEEC CM upregulated PMN phagocytic activity towards sperm (16). Accordingly, in the current study, BEECs were exposed to PGN (10 pg ml^−1^) for 24 h, washed, and co-cultured with sperm for 6 h. Next, sperm-BEEC CM was used to stimulate peripherally-isolated mature PMNs for 2 h and then their inflammatory responses were evaluated. Also, these PMNs were cultured with sperm for 1 h as a phagocytosis assay. Cultivation of PMNs in sperm-BEEC CM sharply increased the expression of various pro-inflammatory genes and *C3* mRNA. However, pre-exposure to PGN blocked these responses and increased the expression of anti-inflammatory genes (*IL10* and *TGFB1*) in PMNs (**Figure 6A**). At the same time, PMN incubation in sperm-BEECs CM increased PMN phagocytic activity for sperm. Conversely, pre-exposure to PGN suppressed sperm phagocytosis by PMNs (**Figures 6B, C** **)**. These results suggest that *the endometrial secretome* obtained from a PGN-sperm-BEEC co-culture could disrupt the innate immune responses of PMNs towards sperm.

### PGN Blocked Sperm-induced Inflammatory Responses in Endometrial Tissues *Ex Vivo*

**Figure 7:**
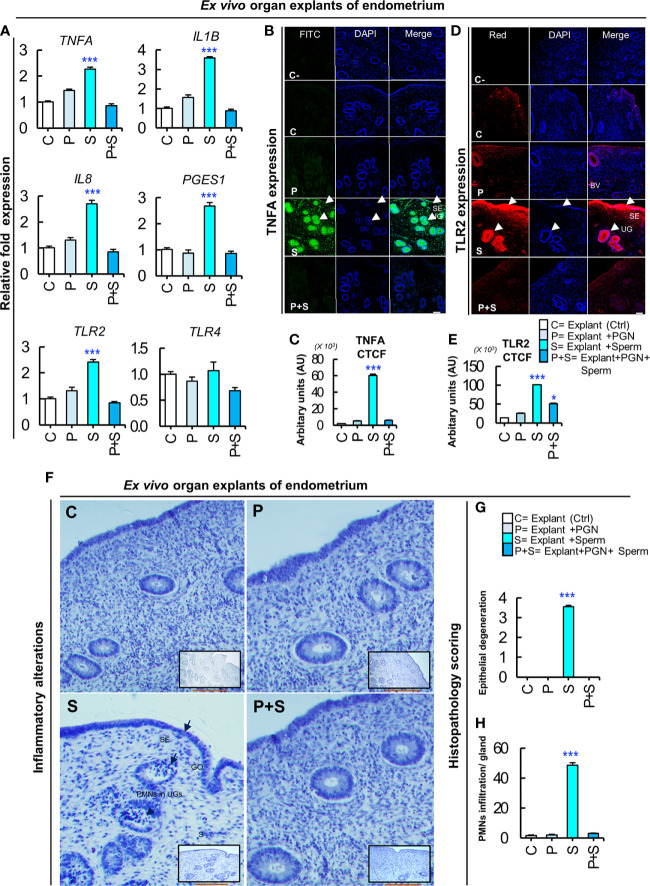
PGN suppresses the sperm-triggered alterations in inflammatory genes and protein expression as well as histomorphology in *ex vivo* explants of endometrium. **(A)** Real-time PCR assay of the mRNA transcription levels of sperm-stimulated inflammatory genes after exposure to PGN. Fresh pre-ovulatory explants were exposed to PGN (10 pg ml^−1^) for 3 h followed by co-culturing with sperm for 3 h. The mRNA expression of different inflammatory genes was assessed. Asterisk denote a significant variance (***P < 0.001) between the different groups when compared to the control group and data are presented as mean ± SEM. **(B)** Immunofluorescence assay of candidate inflammatory marker (TNFA) in *ex vivo* organ explants of endometrium. Immunostaining confirmed intense accumulation of TNFA (green) in UGs and surface epithelium (SE) (arrow heads) compared to stroma (S) in the “S” group and this was abrogated in the “P+S” group. **(C)** Semiquantitative scoring of corrected total cell fluorescence (CTCF) of TNFA was based on mean fluorescence intensity (MFI) and integrated density (IntDen) by *ImageJ* software expressed as arbitrary fluorescence units (AU). DAPI was set as the nuclear counterstain (blue). Normal rabbit IgG replaced the primary antibody in the negative control (C-). **(D)** Immunofluorescence assay of the signaling marker (TLR2) in *ex vivo* organ explants of endometrium. Immunostaining confirmed intense accumulation of TLR2 (red) induced by sperm (“S” group) specifically in UGs and SE (arrow heads), compared to stroma (S) and compared to the C and other treated groups (P, and P+S). **(E)** Semiquantitative scoring of corrected total cell fluorescence (CTCF) of TLR2 expressed as arbitrary fluorescence units (AU). Fluorescence signal was captured using an all-in-one epifluorescence high-resolution imaging system (Keyence, BZ-X800, Osaka, Japan). BZ-X GFP (OP-87763), BZ-X RED (OP-87765), and BZ-X DAPI (OP-87762) filters were set for green, red and blue wavelengths, respectively. Random images from 3 independent experiments (using 3 different healthy pre-ovulatory uteri from 3 different animals) were evaluated and readings were statistically analyzed where asterisks denote a significant variance (*P < 0.05, and ***P < 0.001) between the different groups when compared to the control group and data are presented as mean ± SEM. Magnification: 200×. Scale bar = 50 μm. **(F)** Representative photomicrographs showing delicate inflammatory reaction triggered by sperm in “S” group represented by reversible endometrial tissue injury particularly in uterine glands (UGs), surface epithelium (SE) or gland opening (GO) (short arrows), but not in stroma (S). Hematoxylin and Eosin (H&E) semiquantitative scoring of **(G)** epithelial degeneration, and **(H)** mild neutrophilic infiltration (PMNs) (arrow heads) in UGs was used to quantify degree of histopathological alterations compared to other groups (C, P and P+S) where asterisks denote a significant variance (***P < 0.001) ± SEM. Magnification: 400×. Scale bar = 200 μm.

To further confirm our hypothesis, endometrial explants were exposed to PGN (10 pg ml^−1^) for 3 h followed by co-culturing with sperm for 3 h. The RT-PCR results showed that PGN suppressed the sperm-induced transcription of the tested genes in endometrial explants (**Figure 7A**). The immunofluorescence results showed that co-culturing of sperm with explants increased the mean fluorescence intensity (MFI) of expressed TNFA and TLR2 proteins in the glandular epithelium (GE) and surface epithelium (SE). While, addition of PGN to the sperm-explant co-culture system abrogated MFI of TNFA and TLR2 proteins in these specific sites (**Figures 7B–E**). These findings further confirm a pivotal connection between endometrial responses to PGN and sperm, possibly through the TLR2 pathway.

### PGN Abrogated Sperm-induced Tissue Injury and PMN Recruitment Into Endometrial Glands

Histomorphology of uterine explants revealed that sperm induced a mild degree of reversible tissue injury (epithelial degeneration) in the glandular epithelium and mucous membrane, with marked PMN infiltrations mainly in uterine glands. On the other hand, addition of PGN (10 pg ml^−1^) to the sperm-explant co-culture system completely blocked sperm-induced alterations in the architecture of endometrial tissues (**Figures 7F–H**).

### PGN Increased Sperm Attachment to Endometrial Glands and Surface Epithelium

**Figure 8:**
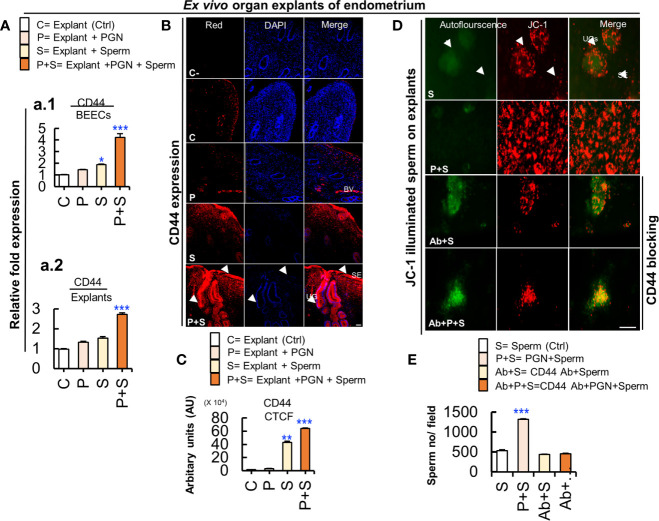
Cluster of differentiation 44 (CD44) is expressed in the bovine endometrium and is implicated in the massive PGN-triggered sperm attachment to endometrial glands and luminal epithelium. **(A)** RT-PCR assay revealed upregulated CD44 mRNA expression in BEECs *in vitro* (a.1) and in endometrial tissue *ex vivo* (a.2). Data are presented as mean ± SEM of 3 independent experiments using epithelial cells or explants from 3 different uteri (3 wells/treatment/experiment). Asterisks denote a significant variance (*P < 0.05, or ***P < 0.001). **(B)** Immunofluorescence assay of cellular adhesion molecule (CD44) in *ex vivo* organ explants of endometrium. Immunostaining confirmed intense accumulation of CD44 (red) (arrow heads) induced by PGN+Sperm (“P+S” group) specifically in UGs and in SE (arrow heads) compared to sperm “S” and other groups (C and P). Magnification: 200×. Scale bar = 50 μm. **(C)** Semiquantitative scoring of corrected total cell fluorescence (CTCF) of CD44 expressed in UGs as arbitrary fluorescence units (AU). Data are presented as mean ± SEM of 3 independent experiments using explants from 3 different uteri (3 wells/treatment/experiment). Asterisks denote a significant variance (**P < 0.01 or ***P < 0.001). **(D)** PGN triggered massive sperm association to uterine glands (UGs) (arrows) and surface epithelium (SE) (arrow heads) compared to control (sperm). Pre-exposure of endometrial explants to anti-rabbit CD44 polyclonal antibody for 3 h followed by exposure to PGN (10 pg ml^−1^) for 3 h, and co-culturing with sperm for 3h (Ab+P+S) resulted in minimizing PGN-triggered sperm attachment (P+S) in endometrial explants and most of spermatozoa were released actively and found freely swimming on the surface epithelium (Ab+PGN+S) (**Supplementary Video 4**). Magnification: 200×. Scale bar = 200 μm. **(E)** ImageJ software (version 1.51j8) was used for counting localized spermatozoa in (**Supplementary Videos 1** and **2**). Data are presented as mean ± SEM of 3 independent experiments using explants from 3 different uteri. Asterisks denote a significant variance (***P < 0.001) between the different groups compared to control (sperm only).

To analyze sperm dynamics, *ex vivo* intact explants of endometrial tissue were exposed to PGN (0 or 10 pg ml^−1^) for 3 h followed by direct co-culture with JC-1–labeled sperm. As early as 5 min, epifluorescence scanning microscopy revealed that vast numbers of spermatozoa clustered mainly in glands with their flagella continuing to beat until after 30 min. Pre-exposure to PGN markedly increased sperm localization in glands, and relatively so in the surface epithelium (**Figure 8D**, and **Supplementary Video 1**, **2**). As well, treatment of explants with pam3Cys (10 pg ml^−1^) triggered sperm attachment to the endometrial epithelium (**Supplementary Video 3**).

### PGN Triggered Sperm Attachment to Endometrial Mucosa *via* Upregulation of Cluster of Differentiation 44 (CD44)

We hypothesize that a CD44 adhesion molecule is involved in PGN-triggered sperm localization to endometrial glands and the surface epithelium. The RT-PCR assay revealed that the combination of PGN with sperm upregulated CD44 mRNA expression in both BEECs and explant models (**Figure 8A**). An immunofluorescence assay confirmed a corresponding increase in CD44 protein expression, especially in glands and surface epithelium (**Figures 8B, C** **)**. Furthermore, addition of anti-CD44 neutralizing antibody minimized sperm attachment to these unique sites and sperm appeared freely moving on the surface epithelium (**Figures 8D, E** **,** and **Supplementary Video 4**). The results suggest that CD44 plays a principal role in PGN-triggered attachment of large numbers of sperm into the endometrial mucosa.

## Discussion

Mucosal surfaces of the female genital tract are the first line of defense against potentially pathogenic microorganisms. Indeed, the uterus is not a completely sterile cavity as it has been classically considered; recently the use of 16S rRNA sequencing provides several lines of evidence that human endometrial mucosa is rich with a Lactobacillus-dominated microbiota (>90% Lactobacillus spp.) and minor alterations in commensal uterine colonization could negatively interfere with local immunity, endometrial receptivity, and fertility (29–31). Our study provides evidence that the presence of traces of pathogen-derived PGN desensitized endometrial mucosa to recognize sperm and thereby preventing sperm-triggered immune responses in the endometrial epithelium mainly *via* the MyD88-dependent pathway of TLR2 signaling. Consequently, the inflammatory response and phagocytic activity of PMNs towards sperm were blocked. Moreover, PGN intensively increased sperm attachment to endometrial glands and surface epithelium *via* the upregulation of a CD44 adhesion molecule. The results demonstrated that during mild pathophysiological conditions, the presence of PGN residues disrupts the transient physiological inflammation induced by sperm in the endometrium epithelium, possibly leading to impairment of uterine clearance and subsequent embryo receptivity and development.

Initiation of innate immune responses through TLRs signaling pathway depends on the nature of the stimulus, stimulation time, and the interaction surfaces on their extracellular domain which activates multiple signaling adaptor proteins through at least two distinct pathways, MyD88-dependent pathway (utilized by all TLRs) and TRIF-dependent pathway (utilized by TLR3 and TLR4 only) (11, 32, 33). Previously, we reported that sperm induced pro-inflammatory responses in BEECs *via* TLR2/4 signaling pathway (17). In the present study, the stimulation of BEECs with high concentrations of TLR4 agonists, LPS, either independently or combined with sperm, induced drastic inflammatory responses in BEECs compared to sperm. Additionally, the pre-exposure of BEECs to low levels of LPS induced slight inflammatory responses in BEECs, but did not interfere with the subsequent sperm-induced inflammation in BEECs. LPS is a highly potent endotoxin (9) which directly binds to the extracellular domain of TLR4, and rapidly stimulates a wide range of downstream signaling cascades (within a few minutes), leading to initiation of a time-dependently progressing inflammatory responses in endometrial cells (17, 34). While, sperm cells trigger weak and transient inflammatory responses in endometrial cells and their interaction with TLR4 domain might be mediated by endogenous ligands (17). Therefore, we suggests that the TLR4-mediated endometrial recognition of sperm or LPS might be generated *via* different intracellular signaling cascades and downstream adaptor proteins. Further investigations are needed to identify different and/or interactive downstream adaptor molecules of TLR4 signaling pathway triggered by sperm or LPS in the endometrial epithelium.

Interestingly, our data showed that low concentrations of TLR2 agonist, PGN, failed to trigger any significant inflammatory responses in BEECs, but they completely blocked sperm-induced inflammation in BEECs, suggesting that there is a pivotal connection between endometrial responses to PGN and sperm, possibly through the TLR2 pathway. However, the ability of high concentrations of PGN to independently induce inflammatory responses in BEECs might mask their suppressive effect on sperm-triggered immune responses in BEECs when combined with sperm. Likewise, the addition of PGN (10 pg ml^−1^) into BEECs culture, primed by luteal stage levels of E2 and P4, followed by co-culturing with sperm, clearly suppressed sperm-induced inflammatory responses in BEECs (**Supplementary Figure 2**), meaning that the suppressive effect of PGN on the sperm-induced inflammation in BEECs was not related to the levels of sex steroids. Moreover, our findings that either pre-exposure or co-exposure of BEECs to PGN (10 pg ml^−1^) blocked the BEECs inflammatory responses to sperm (**Supplementary Figure 3**) indicate that PGN may competitively occupy and block sperm-TLR2 binding and thereby interferes with sperm-triggered immune responses in BEECs (35). This could be attributed to the high affinity of PGN to bind to TLR2 receptors (36). Moreover, the results showed that, in a dose-dependent manner, PGN stimulated the transcription of inflammatory cytokines after 1 h, reached its peak at 3 h, started to subside after 6 h, and completely subsided after 12 to 24 h. Interestingly, the pattern of endometrial inflammation induced by PGN was relatively similar to that induced by the synthetic TLR2 agonist, pam3Cys (17), as well as sperm (16), suggesting again that the TLR2 pathway is involved in the PGN and sperm sensing system in BEECs. Additionally, PGN persistently upregulated transcription levels of *IL10* and *TGFB1* until 12 h and subsided at 24 h. These results clearly show that PGN triggers rapid and transient inflammatory responses in BEECs, which disappear as the amplitude of the inflammation resolves, and reaches baseline after 24 h. By this process, BEECs might undergo a state of inactivation due to relative exhaustion of their inflammatory depot (37), thereby losing their ability to sense and respond to sperm signals through TLR2 (an *autocrine effect)*. Although sperm express TLR2 receptors (38), our data showed that pre-exposure of sperm to PGN did not interfere with the subsequent sperm-induced inflammation in BEECs *in vitro* (**Supplementary Figure 4** **)**, demonstrating that sperm TLR2 are not involved in the suppressive effect of PGN on sperm immune responses in endometrial epithelium.

Immunoblotting analysis revealed that either PGN or sperm stimulated the phosphorylation levels of p38MAPK, ERK1/2, and JNK as downstream targets of MyD88-dependent signaling pathways in BEECs while they did not stimulate the phosphorylation of the IRF3 as downstream targets of TRIF-dependent signaling pathways. This suggests that TLR2 signaling, in particular MyD88-dependent pathway, acts as a commonly-shared pathway for PGN and sperm recognition in BEECs. The results showed that PGN combination with sperm prevented sperm-induced phosphorylation of JNK in BEECs. JNK is a stress-related adaptor protein (39, 40) essential for activation of transcription factor (AP-1) and, consequently, amplification of the inflammatory signal (41). This delicate modulation of TLR2 signaling, induced by PGN, could disturb the entire sperm-triggered TLR2 signal transduction cascade and drive a newly-differentiated inert pathway that impairs the production of inflammatory cytokines/chemokines and dampens BEEC reactivity to sperm (42, 43). Nevertheless, activation or blockage of the TLR2 pathway using its synthetic agonist or antagonist prevented the sperm-induced inflammatory response and PGN effect respectively, which further confirms that BEECs recognize sperm signals mainly *via* the TLR2 pathway. Given that MyD88-dependent pathway is a common pathway for TLR2 and TLR4 (11) together with the fact that TLR2 agonist and antagonist can induce a partial cross-tolerance with TLR4 pathway (44, 45), it seems technically very difficult to clarify the potential contribution of the whole TLR2/4 signaling complex in sperm recognition by endometrial epithelium which deserves further investigations.

Insemination triggers transient physiological inflammatory responses in the endometrium characterized by recruitment of PMNs into the uterine lumen within 2 to 12 h after insemination (14, 46) as the first essential innate immune responses for clearance of bacteria, excess/dead sperm, and tissue debris and these responses enhance endometrial receptivity and pre-implantation embryo development (14). Therefore, sustained or excessive and persistent levels of locally-infiltrated PMNs, as observed during pathophysiological conditions including subclinical endometritis (SE) (47), could interrupt uterine clearance and subsequent embryo receptivity. Our results showed that sperm-BEECs CM induced pro-inflammatory responses in PMNs as *a paracrine effect* of the sperm-endometrial secretome, while pre-exposure to PGN blocked these responses and increased mRNA expressions of anti-inflammatory genes (*IL10* and *TGFB1*). Accordingly, PMNs incubation in sperm-BEECs CM increased PMNs phagocytic activity for sperm while pre-exposure to PGN reduced it. The inhibitory action of pre-exposure to PGN on PMN phagocytosis for sperm might be related to the upregulation of mRNA expression of *IL10* and *TGFB1*, PMN’s phagocytosis-suppressive factors (48), and to the suppression of mRNA expression of *C3*, PMN’s phagocytosis-stimulatory factor (49), in PMNs. Moreover, PGN blocked sperm-induced upregulations of mRNA expressions of *IL8* in PMNs, a strong chemoattractant for PMNs (47). Overall, these results, in part, could explain the molecular mechanism by which the presence of traces of PGN during uterine infection exerts deleterious effects on sperm-endometrial interactions through blocking the physiological endometrial inflammation induced by sperm, thereby preventing the transient PMNs influx and phagocytosis for sperm, which may account for reduced fertility during mild infections. Indeed, it has been shown that the absence of PMNs infiltration into the uterine lumen 4 h after insemination was associated with a reduced conception rate in SE cows (50).

In the same context, using an *ex vivo* intact bovine endometrium explants, we found that PGN clearly blocked sperm-induced inflammatory responses in the endometrial tissue. Additionally, sperm induced a mild degree of reversible tissue injury (epithelial degeneration) in the glandular and surface epithelium with PMNs infiltrations mainly into uterine glands, indicating that the sperm-induced inflammation was not detrimental to the endometrial tissue (51). However, addition of PGN completely blocked sperm-induced alterations in the architecture of endometrial tissues. These observations substantiated our main finding that TLR2 is the central sperm recognition system in endometrial epithelium, specifically in glands. Consequently, disruption of the TLR2 system by PGN could be associated with a nonfunctional mucosal sensing system for sperm, thereby blocking transient inflammatory responses of sperm in the bovine endometrium.

It has been shown that active sperm do not bind to the surface epithelium of the endometrial mucosa, but they enter and are found in the glands (18). Unexpectedly, we found that PGN massively increased attachment of sperm to endometrial glands, and relatively so, to the surface epithelium. In contrast, the pre-treatment of endometrial tissues with LPS did not alter sperm attachment to endometrial epithelium. Given that, PGN can induce CD44 expression through the TLR2 pathway (52) which possesses potent adhesion properties (53) and is expressed in the sperm reservoir to support sperm attachment to the oviductal epithelium (54, 55). Therefore, we hypothesized that CD44 might have a pivotal role in this PGN-triggered sperm attachment to the endometrial epithelium. Our results showed that PGN together with sperm upregulated CD44 expression in uterine glands and surface epithelia. Interestingly, addition of an anti-CD44 neutralizing antibody into a PGN-sperm-explant co-culture decreased sperm attachment into glands and surface epithelia but did not remove the suppressive effect of PGN on the sperm-induced inflammation **(** **Supplementary Figure 5** **)**. Similarly, it has been shown that during fertilization, cumulus cells of ovulated cumulus-oocyte complexes express CD44 to recognize hyaluronan fragments generated from sperm and thereby inflammatory responses in cumulus cells are induced in a TLR2/4-dependent pathway, while addition of a neutralizing antibody for CD44 did not interfere with sperm-triggered inflammation in cumulus cells (56). Previously we reported that, in a dose-dependent manner, sperm induces pro-inflammatory responses in BEECs *in vitro* (16). Therefore, we suggest that PGN blocks the TLR2-mediated sperm sensing system regardless of its ability to increase sperm attachment to the endometrial epithelium *via* the upregulation of CD44 expression in the endometrial tissue. The ability of PGN to trigger sperm attachment with the endometrial epithelium together with its ability to block sperm-induced *PGES1* mRNA expression, which is involved in uterine contractility (57), may impair sperm transport and uterine clearance.

Together, our findings may serve as a proof of concept for mechanisms underlying competitive pathophysiological interactions during either insemination of a mildly infected uterus “pre-exposure” or infection during insemination “co-exposure”. Traces of PGN derived from virtually all bacteria disrupt the sperm sensing system and the subsequently induced inflammatory responses in the bovine endometrium, which in turn might interfere with fertility. The proposed mechanisms involve a desensitized mucosal innate immune system *via* modulation of the MyD88-dependent pathway of TLR2 signaling, inactivation of locally-infiltrating sperm-immune scavengers, PMNs, and triggering sperm attachment to the endometrial tissue, thereby impairing uterine clearance and embryo receptivity. However, apart from our *in vitro* and *ex vivo* investigations, an important next step will be further *in vivo* investigations to understand *in vivo* uterine pathophysiology and its deleterious impact on fertility through, for example, a reduced conception rate.

## Data Availability Statement

The data sets presented in this study can be found in online repositories. The names of the repository/repositories and accession number(s) can be found in the article/**Supplementary Material**.

## Ethics Statement

The animal study was reviewed and approved by The Committee of Animal Experiments at Obihiro University of Agriculture and Veterinary Medicine (Permit no. 27–74).

## Author Contributions

IFE conceived of the study. WG, AAR, MaS, and AM initiated the study design, and MAM, MAZ, IA, TK, MoS, and FN helped with implementation. IFE and MAM provided statistical analysis. All authors contributed to the article and approved the submitted version.

## Funding

This work was supported by Grant-in-Aid for Scientific Research (16H05013, 17F17407, and 20H03122) from Japan Society for the Promotion of Science (JSPS), Japan Association for Livestock New Technology, and Livestock Promotional Funds of Japan Racing Association (JRA). IE has received a scholarship for his PhD program from the Ministry of Higher Education, Egypt.

## Conflict of Interest

The authors declare that the research was conducted in the absence of any commercial or financial relationships that could be construed as a potential conflict of interest.

## Supplementary Material

Supplementary Video 1 JC-1 mitochondrial staining illuminates midpieces of sperm associated with the surface of the endometrial glands. After addition of sperm to preovulatory *ex vivo* organ explants of endometrium, sperm aggregated at the glandular opening after 5 min (and again after 30 min) and (**Figure 7A**). The fluorescence videos were captured using a BZ-X RED (OP-87765) filter and then *ImageJ* software (version 1.51j8) was used for counting of localized spermatozoa. Counts of nine fields, of equal frame size.

Supplementary Video 2 PGN (natural TLR2 ligand) triggered massive sperm association to glands and luminal epithelium in endometrial explants *ex vivo.* Large clusters of motile viable sperm with beating tails accumulated at uterine glands (UDs) and between glands (in the surface epithelium) (SE). The video was captured 5 min (and again after 30 min) after addition of sperm that followed a 3 h pre-exposure to PGN (10 pg ml^−1^).

Supplementary Video 3 pam3Cys (synthetic TLR2 ligand) triggered massive sperm association to glands and luminal epithelium in endometrial explants *ex vivo.* Large clusters of motile viable sperm with beating tails accumulated at uterine glands (UDs) and between glands (in the surface epithelium) (SE). The video was captured 5 min (and again after 30 min) after addition of sperm that followed a 3 h pre-exposure to pam3Cys (10 pg ml^−1^).

Supplementary Video 4 CD44 blocking prevented PGN-triggered sperm association in uterine glands (UDs) and surface epithelium (SE) in endometrial explants *ex vivo.* The video was captured 5 min (and again after 30 min) after addition of sperm that followed a 3 h pre-treatment with CD44 Ab and a 3 h pre-exposure to PGN (10 pg ml^−1^). Most of the spermatozoa that were released form UGs and SE were found freely swimming on the SE showing normal motility and viability.
