# Supplementary material for: Peptidoglycan Switches Off the TLR2-Mediated Sperm Recognition and Triggers Sperm Localization in the Bovine Endometrium
Source: Front Immunol. 2021 Feb 11;11:619408. doi: 10.3389/fimmu.2020.619408 (PMC7905083; doi:10.3389/fimmu.2020.619408)
Supplement: Supplementary file 5 [file DataSheet_1.docx]

Supplementary Material

# Supplementary Figures and Tables

## Supplementary Figures

##
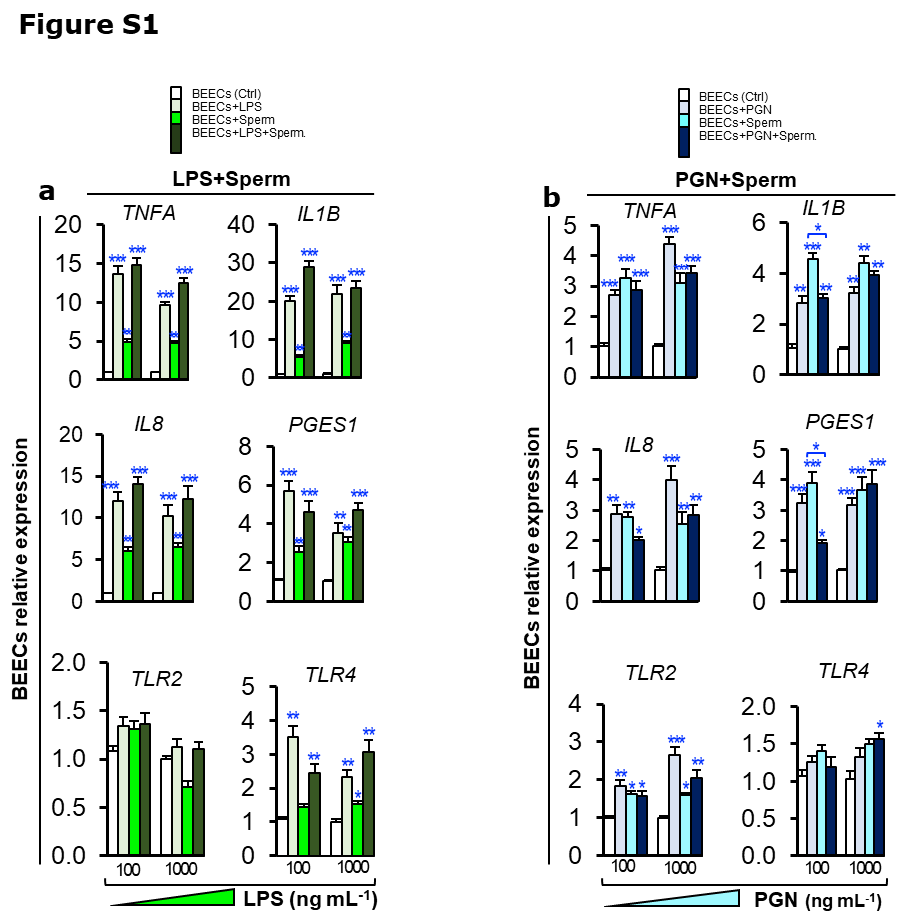


**Supplementary Figure 1**| **High concentrations of LPS or PGN upregulated the transcription of major inflammatory cytokines, chemokine, and PGES1 and their combination with sperm favored the sperm-triggered upregulation of transcription of these genes in BEECs.** Subconfluent BEECs monolayers were exposed to **(a)** LPS (100 and 1000 ng mL^-1^), or **(b)** PGN (100 and 1000 ng mL^-1^) for 24 h followed by co-culturing with sperm (5x10^6^ cell mL^-1^) for 6 h. The mRNA transcription levels of major inflammatory genes (*TNFA, IL1B, IL8, PGES1, TLR2,* and *TLR4*) were quantified by real-time PCR assay. The animal was designated as the statistical unit, and the data was obtained from 3 replicated independent experiments, using BEECs from 3 different uteri, (3 wells/treatment/experiment) presented as mean ± SEM. Asterisks denote a significant variance *(*P<0.05 **P<0.01,* or ****P<0.001)* between the different treatment groups when compared to the control group at each PGN/LPS concentration.


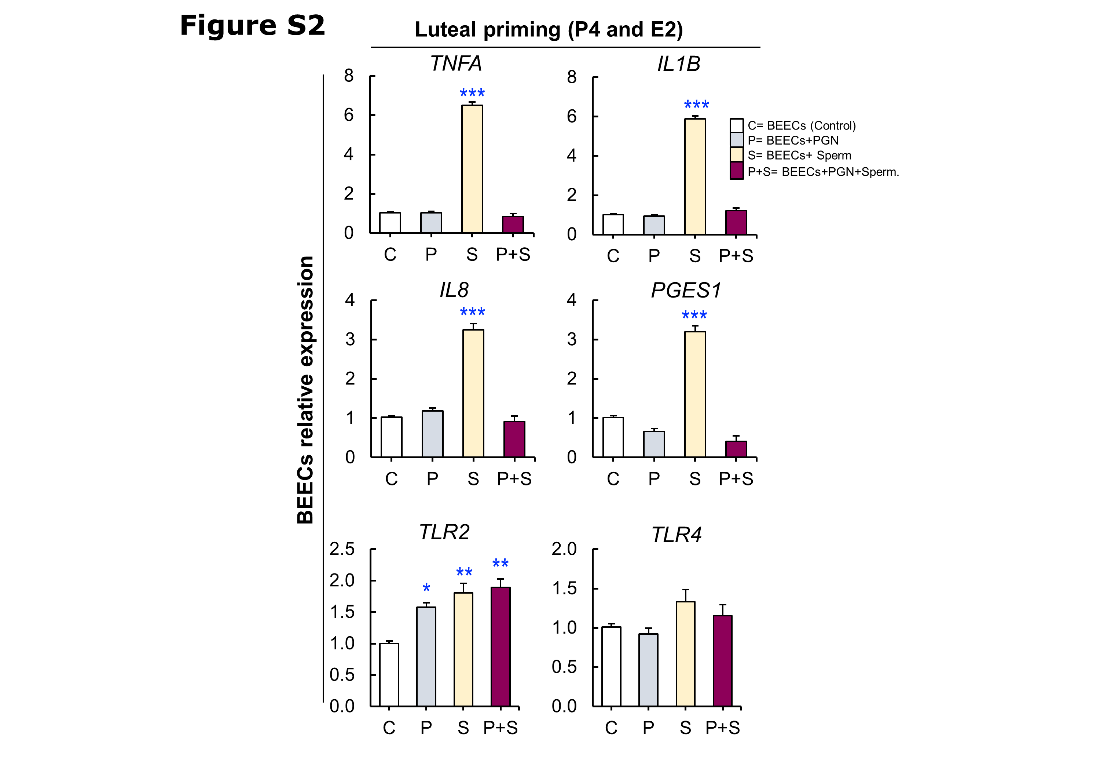


**Supplementary Figure 2** | **PGN suppresses the stimulatory effect of sperm on transcription of major inflammatory genes in BEECs primed with luteal stage levels of P4 and E2 *in vitro*.** BEECs were harvested from luteal uteri, passaged, grown and stimulated under luteal levels of P4 (5 ng mL^-1^) and E2 (3 pg mL^-1^). Subconfluent BEEC monolayers were exposed to PGN (10 pg mL^-1^) for 24 h followed by co-culturing with sperm for 6 h. The mRNA expression of inflammatory transcripts was assessed using RT-PCR assay. Data are presented as mean ± SEM of 3 independent experiments using epithelial cells from 3 different uteri (3 wells/treatment/experiment). Asterisks denote a significant variance *(*P<0.05 **P<0.01, or ***P<0.001*) between the different groups compared to the control.


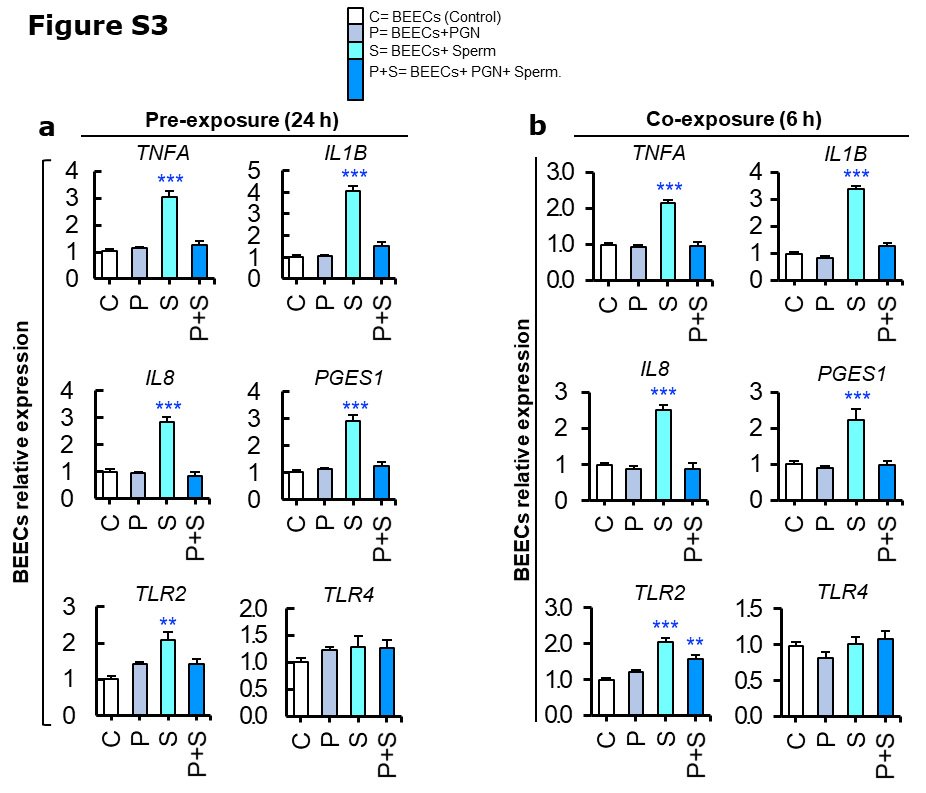


**Supplementary Figure 3** | **(a) Pre-exposure to PGN suppresses the stimulatory effect of sperm on transcription of major inflammatory genes in BEECs.** Subconfluent BEEC monolayers were exposed to PGN (10 pg mL^-1^) for 24 h. Then cells were washed twice and co-cultured with sperm for 6 h. **(b) Co-exposure to PGN for 6 h suppresses the stimulatory effect of sperm on the transcription of major inflammatory genes in BEECs.** Subconfluent BEEC monolayers were exposed to PGN (10 pg mL^-1^) together with sperm for 6 h. The mRNA expression of major inflammatory genes was assessed using RT-PCR assay. Data are presented as mean ± SEM of 3 independent experiments using epithelial cells from 3 different uteri (3 wells/treatment/experiment). Asterisks denote a significant variance *(*P<0.05, **P<0.01, ***P<0.001)* between the different groups when compared to the control group.


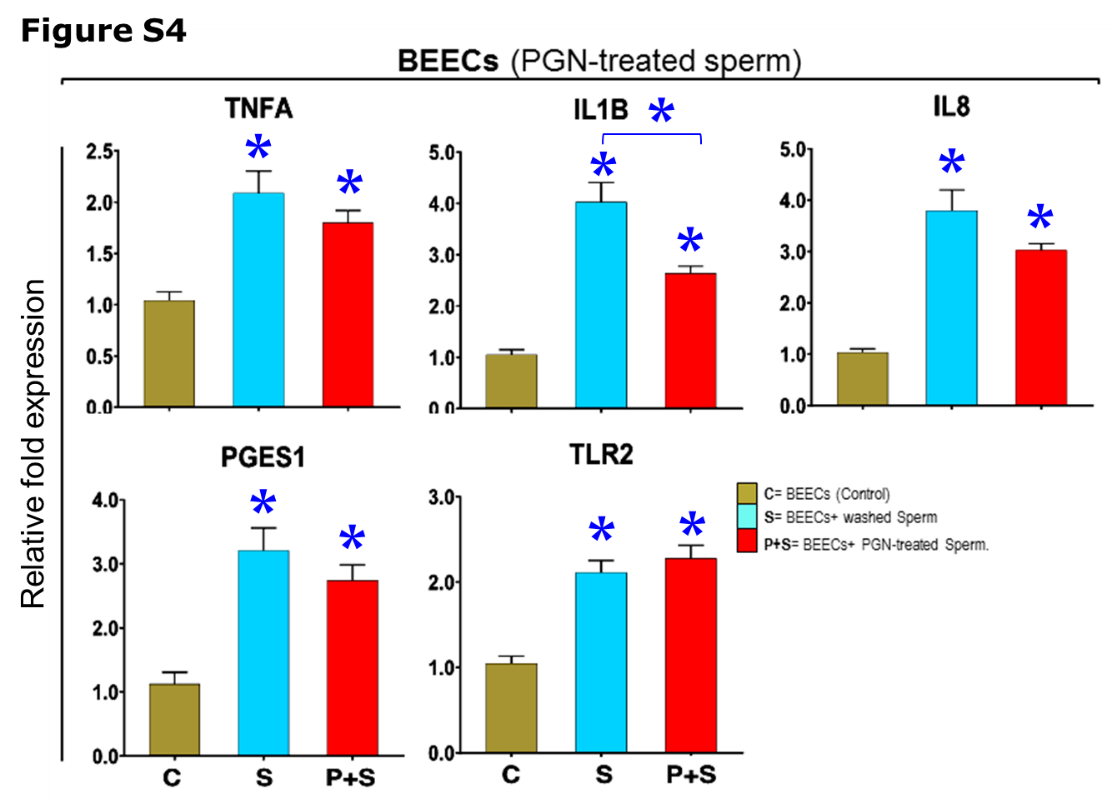


**Supplementary Figure 4** **| PGN-treated sperm upregulated the transcription of major inflammatory cytokines in BEECs *in vitro*.** Bovine sperm (5x10^6^ ml^-1^) were incubated in DMEM/ F12 medium supplemented with PGN (10 pg ml^-1^) for 1 h before co-culturing with BEECs for 6 h. The mRNA expression of inflammatory transcripts was assessed using RT-PCR assay. Data are presented as mean ± SEM of 3 independent experiments using epithelial cells from 3 different uteri (3 wells/treatment/experiment) with significant variance at ANOVA (*P<0.05) between the different groups.


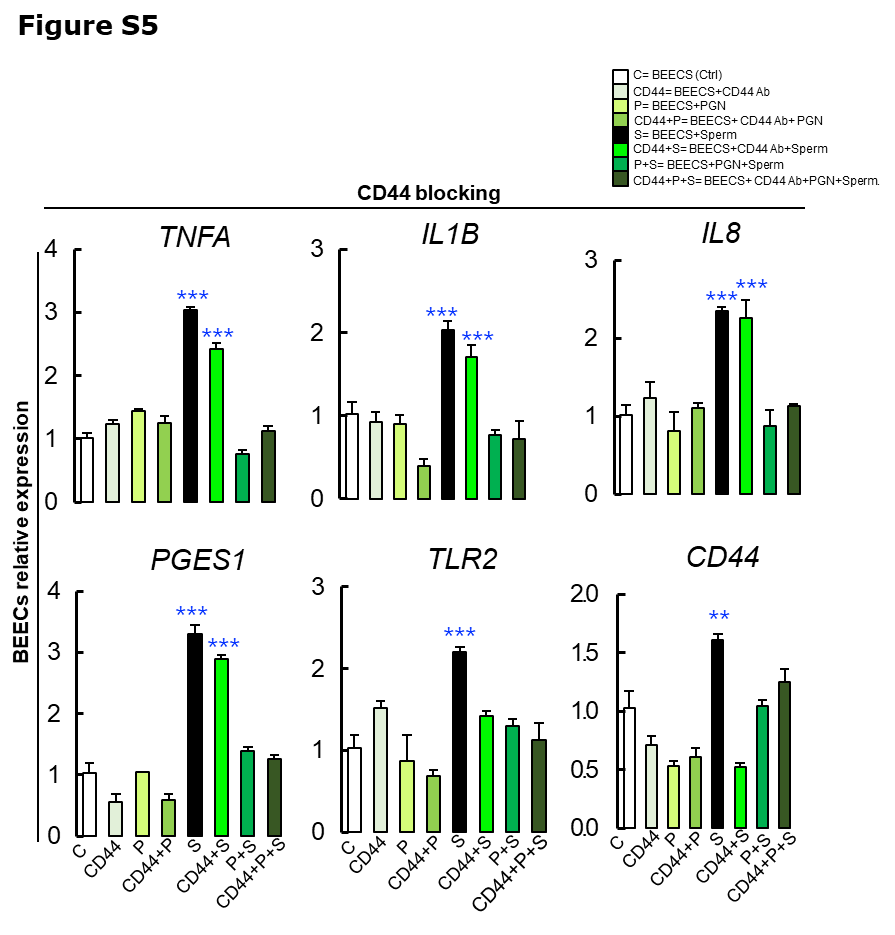


**Supplementary Figure 5** | **CD44 blocking assay did not interfere with the stimulatory effect of sperm on the transcription of major inflammatory genes in BEECs.** Subconfluent BEECs monolayers were pre-exposed to anti-rabbit CD44 polyclonal antibody for 3 h followed by exposure to PGN (10 pg mL^-1^) for 24 h, and then by co-culturing with sperm for 6 h. The mRNA expression of inflammatory transcripts was assessed using RT-PCR assay. Data are presented as mean ± SEM of 3 independent experiments using epithelial cells from 3 different uteri (3 wells/treatment/experiment). Asterisks denote a significant variance (***P<0.01, ***P<0.001)* between the different groups when compared to the control group.

**1.2 Supplementary Tables**

| **Supplementary Table 1\| List of primers used to amplify specific bovine transcripts** | | | | | | | | | |
| --- | --- | --- | --- | --- | --- | --- | --- | --- | --- |
| **Target Gene** | | **Sequence of nucleotide**  **(5' 3')** | **GenBank accession no.** | **Fragment size (bp)** | **Tm (°C)** |  | | |  |
| ***ACTB*** | * F | TCACCAACTGGGACGA CATG | NM_173979.3 | 51 | 60.4 | |  | | |
|  | * R | CGTTGTAGAAGGTGTGGTGCC |  |  | 60.4 | |  | | |
| ***TNFA*** | F | CAAAAGCATGATCCGGGATG | NM_173966.3 | 51 | 56.3 | | |  | |
|  | R | TTCTCGGAGAGCACCTCCTC |  |  | 56.3 | | |  | |
| ***IL1B*** | F | AATCGAAGAAAGGCCCGTCT | NM_174093.1 | 51 | 56.3 | | |  | |
|  | R | ATATCCTGGCCACCTCGAAA |  |  | 56.3 | | |  | |
| ***IL8*** | F | CCAATGGAAACGAGGTCTGC | NM_173925.2 | 51 | 58.6 | | |  | |
|  | R | CCTTCTGCACCCACTTTTCCT |  |  | 56.3 | | |  | |
| ***PGES1*** | F | AAAATGTACGTGGTGGCCGT | NM_174443.2 | 51 | 56.3 | | |  | |
|  | R | CTTCTTCCGCAGCCTCACTT |  |  | 58.4 | | |  | |
| ***TLR2*** | F | CATGGGTCTGGGCTGTCATC | NM_174197.2 | 51 | 60.4 | | |  | |
|  | R | CCTGGTCAGAGGCTCCTTCC |  |  | 62.5 | | |  | |
| ***TLR4*** | F | CCCCTTTCAGCTCTGCCTTC | NM_174198.6 | 51 | 60.4 | | |  | |
|  | R | ATGGCCACCCCAGGAAT |  |  | 54.0 | | |  | |
| ***NFκB2*** | F | CCTGCTGAATGCTCTGTCTG | NM−001102101.1 | 102 | 58.4 | | |  | |
|  | R | TCCTCCTTCACCTCTGTGCT |  |  | 58.4 | | |  | |
| ***IL10*** | F | GAGATGCGAGCACCCTGTCT | NM−174088.1 | 51 | 60.4 | | |  | |
|  | R | GGCTGGTTGGCAAGTGGATA |  |  | 58.4 | | |  | |
| ***TGFB1*** | F | CTTTCTTCAAATGCAGCATTGG | NM−001166068 | 90 | 58.8 | | |  | |
|  | R | GGGTCTGGGTGATACAACGAA |  |  | 60.4 | | |  | |
| ***C3*** | F | TTGGAGAAGCGGCAGGAGTC | AM086793 | 51 | 60.4 | | |  | |
|  | R | TATTGGAAGGCGGCGTAGGC |  |  | 60.4 | | |  | |
| ***Casp 3*** | F | TGTGCTTCTAAGCCATGGTG | BC123503.1 | 51 | 56.3 | | |  | |
|  | R | TCTGCAATAGTCCCCTCTGAA |  |  | 56.5 | | |  | |
| *ACTB:* bovine beta-actin (housekeeping), *TNFA:* tumor-necrosis factor-alpha, *IL1B:* interleukin 1 beta, *IL8:* interleukin 8, *PGES1:* prostaglandin E synthase-1, *TLR2:* toll-like receptor 2, *TLR4:* toll-like-receptor 4, *NFκB2:* nuclear factor kappa B, *IL10:* interleukin 10, *TGFB1:* transforming growth factor 1, *C3:* complement component 3, *Casp3:* bovine caspase 3, * F: forward, *R: reverse. | | | | | | | | | |

| **Supplementary Table 2\| List of antibodies used for immunoblotting** | | | | |
| --- | --- | --- | --- | --- |
| **Target** | **Antibody** | **Source (Catalog no.)** | **Species** | |
| **Phospho p38 MAPK** | Anti-phospho-p38 MAP kinase antibody | Cell signaling (9211S) | Rabbit | |
| **p38 MAPK** | Anti-p38 MAP kinase (C-20) antibody | Santa Cruz Biotechnology (sc-535) | Goat | |
| **Phospho ERK1/2** | Anti-phospho-p44/42 MAPK (Erk1/2) (Thr202/Tyr204) (20G11) antibody | Cell signaling (4376S) | Rabbit | |
| **ERK1/2** | Anti- p44/42 MAPK (Erk1/2) (L34F12) antibody | Cell signaling (4696S) | Mouse | |
| **phospho JNK** | Anti-phospho-SAPK/JNK (Thr183/Tyr185) antibody | Cell signaling (9251) | Rabbit | |
| **JNK** | Anti-JNK (FL) antibody | Santa Cruz Biotechnology (sc-571) | Rabbit | |
| **Phospho NFκB** | Anti-phospho-NFκB p65 (Ser536) (93H1) antibody | Cell signaling (3033S) | Rabbit | |
| **NFκB** | Anti-NF-κB p65 (D14E12) antibody | Cell signaling (8242S) | Rabbit | |
| **Phospho IRF3** | Anti-phospho-IRF3 (S396)(4D4G) antibody | Cell signaling (4947S) | Rabbit | |
| **β-actin** | Anti-β-actin monoclonal antibody | Sigma-Aldrich (A5316) | Mouse | |
| All primary antibodies in western blotting used 1:1000 dilutions, secondary antibodies for beta-actin (β-actin) and total ERK 1/2 used 1:5000 dilutions, and other secondary antibodies used 1:3000 dilutions. | | | | |

| **Supplementary Table 3\| List of antibodies used for immunofluorescence (IF)** | | | | | |
| --- | --- | --- | --- | --- | --- |
| **Target** | **Antibody** | **Source**  **(Catalog no.)** | **Dilution** | |  |
| **TNFA** | Mouse monoclonal anti-bovine TNFA primary antibody | BIO-RAD (MCA2334) | 1:200 |  | |
| **TLR2** | Rabbit polyclonal anti-TLR2 primary antibody | Biorybt (ORB11487) | 1:50 |  | |
| **CD44** | Rabbit polyclonal anti-CD44 primary antibody | Pgtlab (15675-1-AP) | 1:50 |  | |
| **IgG** | Goat anti-mouse IgG secondary antibody | Invitrogen  (A-11029) | 1:200 |  | |
| **IgG** | Goat anti-rabbit IgG secondary antibody | Invitrogen  (A-11035) | 1:200 |  | |
| Target retrieval solution (1:10, S1699, citrate buffer at pH 7.5). | | | | | |

# 2. Supplementary Videos

**Supplementary Video 1| JC-1 mitochondrial staining illuminates midpieces of sperm associated with the surface of the endometrial glands.** After addition of sperm to preovulatory *ex vivo* organ explants of endometrium, sperm aggregated at the glandular opening after 5 min (and again after 30 min) and (**Fig. 7a**). The fluorescence videos were captured using a BZ-X RED (OP-87765) filter and then *ImageJ* software (version 1.51j8) was used for counting of localized spermatozoa. Counts of nine fields, of equal frame size.

**Supplementary Video 2| PGN (natural TLR2 ligand) triggered massive sperm association to glands and luminal epithelium in endometrial explants *ex vivo.*** Large clusters of motile viable sperm with beating tails accumulated at uterine glands (UDs) and between glands (in the surface epithelium) (SE). The video was captured 5 min (and again after 30 min) after addition of sperm that followed a 3 h pre-exposure to PGN (10 pg mL^-1^).

**Supplementary Video 3| pam3Cys (synthetic TLR2 ligand) triggered massive sperm association to glands and luminal epithelium in endometrial explants *ex vivo.*** Large clusters of motile viable sperm with beating tails accumulated at uterine glands (UDs) and between glands (in the surface epithelium) (SE). The video was captured 5 min (and again after 30 min) after addition of sperm that followed a 3 h pre-exposure to pam3Cys (10 pg mL^-1^).

**Supplementary Video 4| CDD4 blocking prevented PGN-triggered sperm association in uterine glands (UDs) and surface epithelium (SE) in endometrial explants *ex vivo.*** The video was captured 5 min (and again after 30 min) after addition of sperm that followed a 3 h pre-treatment with CD44 Ab and a 3 h pre-exposure to PGN (10 pg mL^-1^). Most of the spermatozoa that were released form UGs and SE were found freely swimming on the SE showing normal motility and viability.
